# IL-17 Inversely Correlated with IL-10 via the STAT3 Gene in *Pneumocystis*-Infected Mice

**DOI:** 10.1155/2019/6750861

**Published:** 2019-09-10

**Authors:** Heng-Mo Rong, Xiao-Jun Qian, Chao Zhang, Ting Li, Zhao-Hui Tong

**Affiliations:** ^1^Department of Respiratory and Critical Care Medicine, Beijing Institute of Respiratory Medicine and Beijing Chaoyang Hospital, Capital Medical University, Beijing, China; ^2^Department of Respiratory Medicine, The Third People's Hospital of Hefei, Hefei, China

## Abstract

**Background:**

*Pneumocystis* pneumonia (PCP) remains a common opportunistic infection in immunosuppressed individuals. Current studies showed that multiple immune cells and cytokines took part in the host defense against *Pneumocystis* (PC). However, the roles of IL-17 and IL-10 in the development of PCP have not been elucidated.

**Methods:**

IL-10 and IL-17 levels in serum from PCP mice were detected via ELISA. The percentages of B10 cells, IL-10^+^ macrophages, and IL-10^+^ T cells in the lung from IL-17^–/–^ PCP mice and Th17 cells and IL-17^+^*γδ*T cells in IL-10^–/–^ PCP mice were examined via flow cytometry. Also, antibody neutralization examination was also performed to elucidate the relationship of IL-17 and IL-10 in the PCP model.

**Results:**

We noted the increase of IL-17 and IL-10 levels in serum from mice infected with *Pneumocystis*. Furthermore, deficiency of IL-17 or IL-10 could lead to the delayed clearance of *Pneumocystis* and more severed lung damage. Our data also demonstrated that IL-17 deficiency enhanced the serum IL-10 level and the percentages of B10 cells, IL-10^+^ macrophages, and IL-10^+^ T cells in the lung from PCP mice. Interestingly, we also noted an increase of the IL-17 level in serum and Th17 cell and IL-17^+^*γδ*T cell percentages in the lung from IL-10^–/–^ PCP mice. Using antibody neutralization experiments, we found that the STAT3 gene might play a critical role in the interplay of IL-17 and IL-10 in PCP.

**Conclusion:**

Taken together, our results demonstrated that IL-17 and IL-10 could play the protective roles in the progression of PCP and the inverse correlation of them might be mediated by STAT3.

## 1. Introduction

*Pneumocystis* pneumonia (PCP) is the leading cause of lung infections in HIV-positive individuals worldwide [1, 2]. Recently, newer use of immunosuppressive agents and chemotherapeutics on patients with autoimmune conditions, transplantation, and hematologic malignancies lends to the development of PCP. In addition, HIV-negative PCP hosts tend to have a higher mortality rate and have a more fulminant presentation with substantial dyspnea, fever, and chills. Furthermore, HIV-negative patients are more likely to require mechanical ventilation [3–6]. The immune system can mount a pathologic response against *Pneumocystis* and result in severe damage to the host lung. Recent studies have demonstrated that multiple immune cells and cytokines participate in the development of PCP. These include macrophages, Th1 cells, Th2 cells, Th17 cells, B cells, and the other immune cells. However, the pathogenesis of PCP has not been elucidated.

The alveolar macrophages (AMs) are the first line of host defense to *Pneumocystis*. The critical role of AMs lies in their capability to directly kill both trophozoites and cysts, leading to adaptive immune responses [7, 8]. CD4^+^ T cells are demonstrated to play a critical role in memory cell functions via recruiting and activating the effector cells [9]. Several studies suggested that Th1, Th2, and Th17 cells could play the protective roles in host inflammatory responses. Mounting IFN-*γ* could attenuate the lung damage of the *Pneumocystis*-infected rat [10]. Th2 cell deficiency leads to the persistent eosinophilic infiltration in PCP mice [11]. An increase of Th17 cells was noted in PCP hosts; however, Ripamonti et al. found that IL-17 could not help to eliminate the *Pneumocystis* cysts [12, 13]. Nowadays, accumulating evidence indicates that B cells might play a vital role of promoting the proliferation and activation of CD4^+^ T cells during *Pneumocystis* infection [14]. Our previous study also demonstrated that B10 cells regulated the Th1/Th17 cell immune responses in the PCP model [15].

IL-17 is a tissue-signaling cytokine that favors protection of barrier organs such as the skin, lung, and gastrointestinal system [16]. It is one of the critical proinflammatory cytokines and related to multiple diseases [17, 18]. IL-17 was secreted by Th17 cells, *γδ*T cells, iNKT cells, and group 3 ILCs [19]. IL-10 is one of the most significant anti-inflammation cytokines produced during infectious diseases and cancer [20]. During *Pneumocystis* infection, IL-10 was demonstrated to play a protective role in reducing the immune response to pathogen, alleviating lung damage, and mediating B cell protection-demand hematopoiesis in PCP hosts [21, 22]. Several studies have demonstrated that IL-10 could inhibit immune responses in multiple diseases [23–25]. However, the roles of IL-17 and IL-10 in PCP hosts have not been clearly elucidated.

In this study, we focused on the functions of IL-17 and IL-10 and their interactions in *Pneumocystis*-infected individuals.

## 2. Materials and Methods

### 2.1. Mice

Wild-type (WT) C57BL/6 mice and severe combined immunodeficient (SCID) mice were purchased from Beijing Vital River Laboratory Animal Technology Co. Ltd. (Beijing, China). IL-10^–/–^ mice (stock no. 002251) with the C57/BL6 background were purchased from The Jackson Laboratory (Bar Harbor, ME, USA). IL-17^–/–^ mice on a C57BL/6 background were provided by Dr. Iwakura (University of Tokyo, Tokyo, Japan). Mice used for experiments were 6-8 wk females. They were bred on a chow diet in ventilated cages in the Animal Care Facility of Beijing Chaoyang Hospital. All of the animal studies were approved by the Capital Medical University Animal Care and Use Committee.

### 2.2. PCP Models and Sample Processing

*Pneumocystis murina* was maintained in CB17 SCID mice, and lung homogenates were used to get *Pneumocystis* cysts as previously described [15, 26]. After the lung homogenates were stained using Diff-Quick (Baxter, McGaw Park, IL), the number of *Pneumocystis* cysts was determined microscopically. PCP models were prepared by intratracheally inoculating with 1 × 10^6^ cysts in 100 *μ*l of PBS. Mice were sacrificed at serial time postinfection. Periodic acid silver methenamine staining of the lung was used to confirm *Pneumocystis* infection (Supplementary Fig. [Supplementary-material supplementary-material-1]). *Pneumocystis* burden in the lung was detected by real-time PCR as previously described. Primers and probes for the *P*. *murina* RNA were described in the online supplement.

### 2.3. Flow Cytometry

Cells from tissue and blood were stained with innate cell-specific, B cell-specific, and T cell-specific panels, as described previously [15], and analyzed using FACSCanto II (BD Biosciences, San Jose, CA, USA). The antibody panel is described in the online supplement.

### 2.4. Real-Time PCR

mRNA expression of STAT3, STAT5, ROR*γ*T, IFN-*γ*, STAT1, GATA3, and Irf4 in the lung from infected mice was determined by real-time PCR (RT-PCR). Primers and probes were described in the online supplement.

### 2.5. Enzyme-Linked Immunosorbent Assay

Blood from PCP patients and mice was centrifuged at 1,000 g to obtain sera. IL-10 and IL-17A in serum samples were detected using ELISA Kits (eBioscience, San Diego, CA, USA) according to the manufacturer's instructions.

### 2.6. IL-17 and IL-10 Neutralization In Vivo

C57BL/6 mice were inoculated intraperitoneally twice weekly with 200 *μ*g of anti-mouse IL-17A clone 17F3 (Bio X cell, West Lebanon, NH) [27] or 200 *μ*g of anti-mouse IL-10 clone 1B1.3A (Bio X cell, West Lebanon, NH) [28]. The control group received an equal volume of PBS. Mice were sacrificed at 2 wk postinfection.

### 2.7. Statistical Analysis

Statistical analysis was performed using Prism 5.0 (GraphPad Software, San Diego, CA). Data were described as mean ± SEM. We performed statistical analysis by Student's *t*-test for two-group comparison. All hypothesis tests were conducted at the 0.05 level of statistical significance.

## 3. Results

### 3.1. IL-17A/IL-10 Levels Increased in *Pneumocystis*-Infected Mice

**Figure 1:**
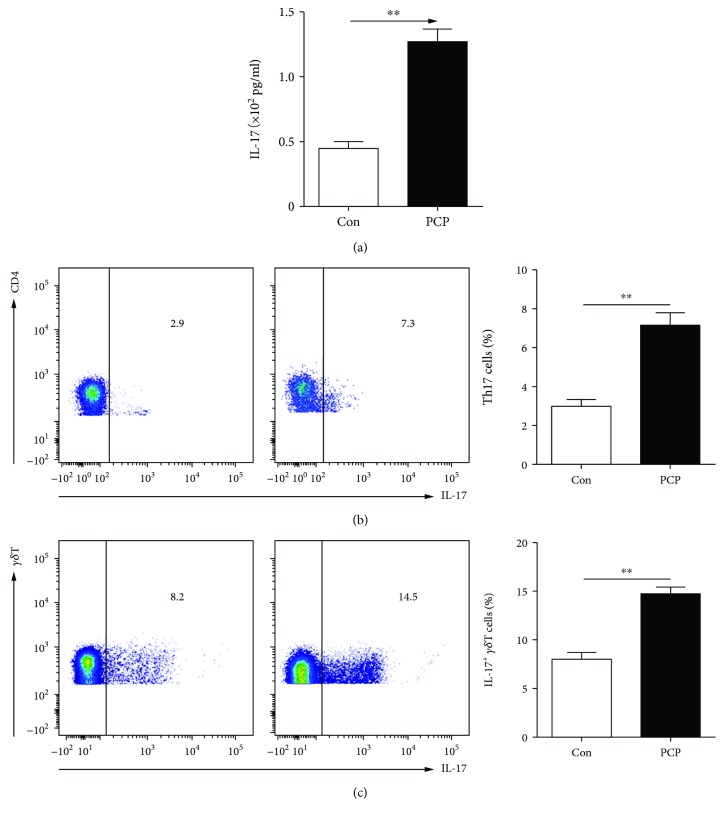
IL-17 levels increased in *Pneumocystis*-infected mice. IL-17 levels in the sera of PCP mice and WT mice were examined via ELISA (a). Representative flow cytometric dot plots and comparisons of Th17 (CD4^+^IL-17^+^) cells (b) and IL-17^+^*γδ*T (*γδ*T^+^IL-17^+^) cells (c) in the lungs from PCP mice and WT mice. Comparisons were evaluated by Student's *t*-tests for two-group comparisons. ^∗∗^*P* < 0.01 and ^∗∗∗^*P* < 0.001. Con: control; PCP: *Pneumocystis* pneumonia.

ELISA data demonstrated a significant increase of the IL-17A level in PCP mice compared with that in the corresponding serum from WT mice (1.25 ± 0.18 × 10^2^ vs 0.45 ± 0.05 × 10^2^ pg/ml, *P* < 0.01, Figure 1(a)). Also, the percentages of Th17 cells increased in the lung from *Pneumocystis*-infected mice than those from uninfected mice (7.50 ± 0.15 vs 3.07 ± 0.36%, *P* < 0.01, Figure 1(b)). In addition, *γδ*T cells from PCP mice were expressing more IL-17A than those from WT mice (14.2 ± 0.18 vs 8.5 ± 0.12, *P* < 0.01, Figure 1(c)).

**Figure 2:**
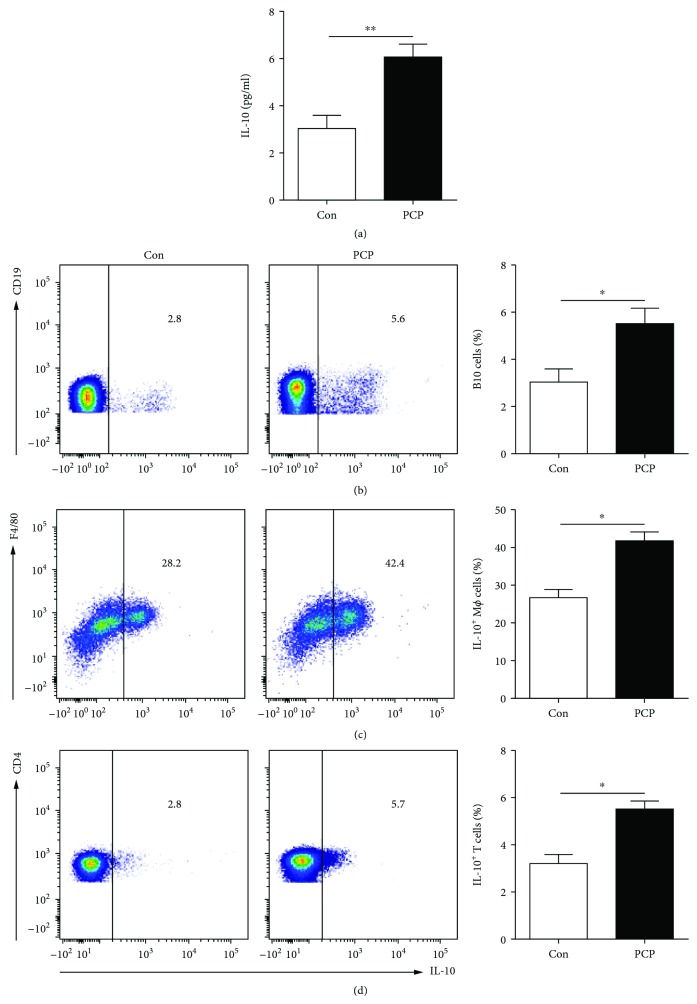
IL-10 levels increased in *Pneumocystis*-infected mice. The levels of IL-10 in the serum of PCP mice (a) were examined by ELISA. Representative flow cytometric dot plots and comparisons of B10 cells (CD19^+^IL-10^+^) (b), IL-10^+^ macrophages (F4/80^+^IL-10^+^) (c), and IL-10^+^CD4^+^ T cells (CD4^+^IL-10^+^) (d) in the lung from PCP mice and WT mice. Comparisons were evaluated by Student's *t*-tests for two-group comparisons. ^∗^*P* < 0.05. Con: control; PCP: *Pneumocystis* pneumonia.

We also noted that IL-10 concentrations in the serum from PCP mice were higher than those from WT mice (5.9 ± 0.2 vs 3.0 ± 0.3 pg/ml, *P* < 0.01, Figure 2(a)). FACS data showed the significant increase of IL-10-producing B cell (5.7 ± 0.4 vs 2.9 ± 0.6%, *P* < 0.05, Figure 2(b)), macrophage (43.5 ± 2.5 vs 29.3 ± 1.8%, *P* < 0.05, Figure 2(c)), and T cell (5.8 ± 0.9 vs 2.9 ± 1.2%, *P* < 0.05, Figure 2(d)) percentages in the lung from *Pneumocystis*-infected mice than those from uninfected mice. Furthermore, we detected the percentages of IL-17- and IL-10-expressing mononuclear cells in blood from mice. The results demonstrated that there were few IL-17-producing cells and IL-10-producing cells in blood from mice. Also, we did not note significant differences of the percentages of these cells in blood from PCP mice and WT mice (Supplementary Fig. [Supplementary-material supplementary-material-1]). Meanwhile, the PCP model was built by intratracheally inoculating with cysts and severe infection was observed in the lung of mice in our previous study [15]. According to these results, we focused on the immune cells in the lung from mice after *Pneumocystis* infection in the next experiments.

### 3.2. IL-17 and IL-10 Were Associated with the Clearance of *Pneumocystis* Cysts

**Figure 3:**
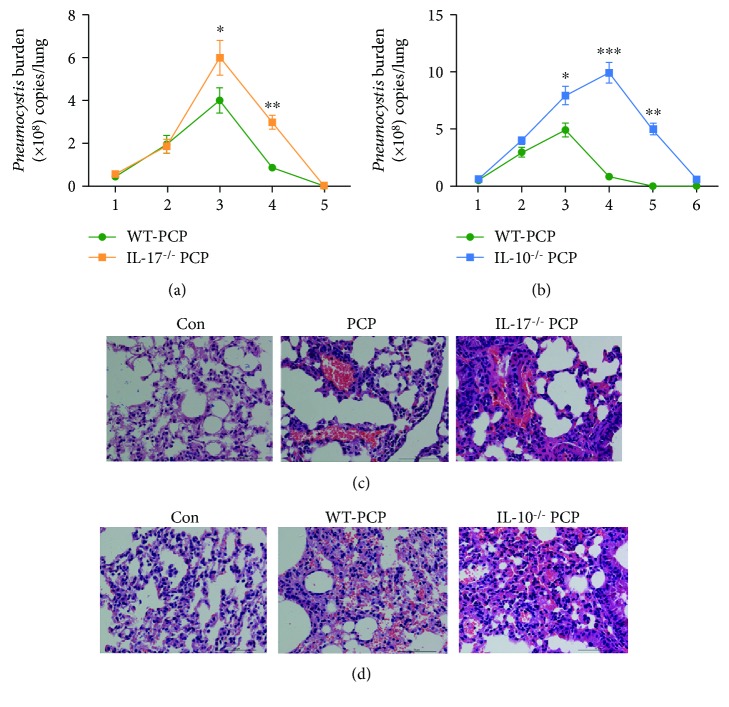
IL-17 and IL-10 were associated with the clearance of *Pneumocystis* cysts. Comparisons of *Pneumocystis* lung burden in the lungs from WT PCP mice (*n* = 5) and IL-17^–/–^ mice (*n* = 5) (a). Comparisons of *Pneumocystis* lung burden in the lungs from WT PCP mice (*n* = 5) and IL-10^–/–^ mice (*n* = 5) (b). H&E-stained histological features of the lungs in WT mice and IL-17^–/–^ PCP mice (c). H&E-stained histological features of the lungs in WT mice and IL-10^–/–^ PCP mice (d). In (a, b), the results are presented as means ± SE of 5 mice per group in each experiment, performed in triplicate at different time points. ^∗^*P* < 0.05, ^∗∗^*P* < 0.01, and ^∗∗∗^*P* < 0.001. Comparisons were evaluated by Student's *t*-test for two-group comparisons. Con: control; H&E: hematoxylin-eosin; PCP: *Pneumocystis* pneumonia.

IL-17^–/–^ mice and IL-10^–/–^ mice were used to investigate the roles of IL-17 and IL-10 in the clearance of *Pneumocystis*. *Pneumocystis*-infected IL-17^–/–^ and IL-10^–/–^ mice were sacrificed at 1-5 wk postinfection. Using RT-PCR, we found that after 3 wk postinfection, *Pneumocystis* burden in WT mice started to decrease. However, IL-17^–/–^ mice and IL-10^–/–^ mice showed delayed clearance of *Pneumocystis* in the lung (Figures 3(a) and 3(b)). We performed hematoxylin and eosin (H&E) staining of the lung homogenates from IL-17^–/–^ PCP mice and IL-10^–/–^ PCP mice at 2 wk postinfection. Compared with WT PCP mice, IL-17^–/–^ PCP mice and IL-10^–/–^ PCP mice showed more severe alveolar hemorrhage and inflammation cell infiltration in the lung (Figures 3(c) and 3(d)).

### 3.3. IL-17 and IL-10 Inversely Correlated with Each Other in *Pneumocystis*-Infected Mice

**Figure 4:**
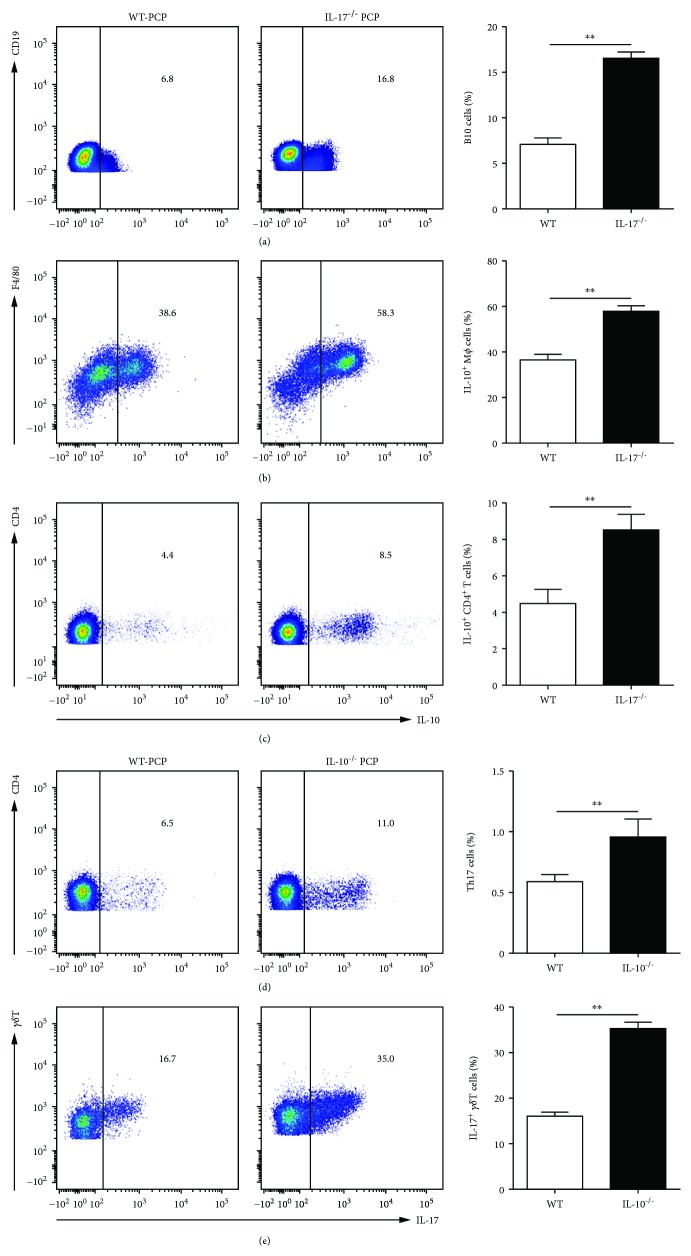
IL-17 inversely interplayed with IL-10 in the PCP model. Representative flow cytometric dot plots and comparisons of B10 cells (CD19^+^IL-10^+^) (a), IL-10^+^ macrophages (F4/80^+^IL-10^+^) (b), and IL-10^+^ T cells (CD4^+^IL-10^+^) (c) in the lungs from WT PCP mice and IL-17^–/–^ PCP mice. Representative flow cytometric dot plots and comparisons of Th17 cells (CD4^+^IL-17^+^) (d) and IL-17^+^*γδ*T cells (*γδ*T^+^IL-17^+^) (e) in the lungs from WT PCP mice and IL-10^–/–^ PCP mice. ^∗∗^*P* < 0.01. Comparisons were evaluated by Student's *t*-test for two-group comparisons. WT: wild type; PCP: *Pneumocystis* pneumonia.

To further explore the role of IL-17 in *Pneumocystis* infection, we detected the percentages of B cells, T cells, and macrophages in the lungs from IL-17^–/–^ PCP mice and WT PCP mice at 2 wk postinfection. The results did not show significant differences of the percentages of these cells between WT PCP mice and IL-17^–/–^ PCP mice (Supplementary Fig. [Supplementary-material supplementary-material-1]). However, flow cytometry data showed the significant increase of IL-17-producing B cells (16.9 ± 1.5 vs 6.7 ± 1.2%, *P* < 0.01, Figure 4(a)), macrophages (58.5 ± 2.4 vs 39.5 ± 1.9%, *P* < 0.01, Figure 4(b)), and T cells (8.5 ± 0.2 vs 4.4 ± 0.1%, *P* < 0.01, Figure 4(c)) in the lung from IL-17^–/–^ PCP mice than those from WT PCP mice. Also, the percentages of T cells, B cells, and *γδ*T cells were detected in WT-PCP mice and IL-10^–/–^ PCP mice and we noted the decreased B cells and increased *γδ*T cells in IL-10^–/–^ PCP mice (Supplementary Fig. [Supplementary-material supplementary-material-1]). Next, we performed experiments to investigate if IL-10 influences the production of IL-17 in the PCP model. Similar to what we found in IL-17^–/–^ PCP mice, we noted that CD4^+^ T cells (10.0 ± 1.5 vs 6.5 ± 0.9%, *P* < 0.01, Figure 4(d)) and *γδ*T cells (35.2 ± 2.1 vs 16.5 ± 1.6%, *P* < 0.01, Figure 4(e)) were expressing more IL-17 in the lung from IL-10^–/–^ PCP mice than WT PCP mice.

### 3.4. IL-17-Related Gene Expression in PCP Mice

**Figure 5:**
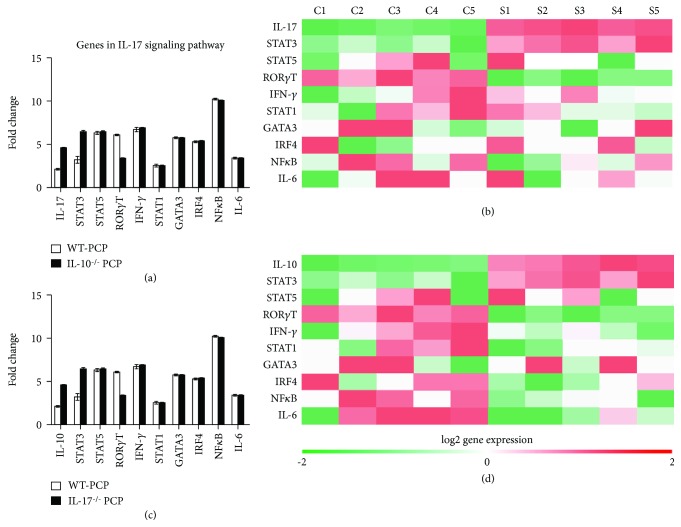
IL-17-related gene expression in IL-10^–/–^ PCP mice and IL-17^–/–^ PCP mice. Genes in the IL-17 signaling pathway from the lungs of WT PCP mice and IL-10^–/–^ PCP mice were examined by RT-PCR (a), and the expression of genes were shown in the heat map (b). IL-10 and genes in the IL-17 signaling pathway from the lungs of WT PCP mice and IL-17^–/–^ PCP mice were examined by RT-PCR (c), and the expression of genes was demonstrated in the heat map (d). Comparisons were evaluated by Student's *t*-test for two-group comparisons. WT: wild type; PCP: *Pneumocystis* pneumonia.

Since IL-17-expressing B cells, macrophages, and T cells were significantly increased in the lung from IL-10^–/–^ PCP mice, we explored whether IL-10 would make an impact on IL-17-related genes. RT-PCR data demonstrated that IL-17 and STAT3 gene expression was significantly increased in the lung from IL-10^–/–^ PCP mice than that from WT PCP mice at 2 wk after *Pneumocystis* infection. The expression of ROR*γ*T was downregulated in IL-10^–/–^ PCP mice. There were no significant differences of the other related genes such as STAT5, STAT1, GATA3, IFN-*γ*, IRF4, NF*κ*B, and IL-6 between IL-10^–/–^ PCP mice and WT PCP mice (Figures 5(a) and 5(b)). The above data indicated that IL-10 deficiency might promote IL-17 expression via the STAT3 gene.

Next, we elucidated the change of IL-10 expression and IL-17-related genes in the lung of IL-17^–/–^ PCP mice. Our data demonstrated that IL-10 and the STAT3 gene were upregulated in the lung from IL-17^–/–^ PCP mice compared with WT-PCP mice after 2 wk of infection with *Pneumocystis* (Figure 5(c)). However, ROR*γ*T, STAT1, IRF4, IL-6, and IFN-*γ* genes were downregulated in IL-17^–/–^ PCP mice. Furthermore, there was no significant difference of the other genes between IL-17^–/–^ PCP mice and WT PCP mice. Thus, STAT3 may play an important role in the interplay of IL-10 and IL-17 in the *Pneumocysti*s-infected mouse model.

### 3.5. STAT3 Played a Role in the Interplay of IL-17 and IL-10 in the PCP Model

**Figure 6:**
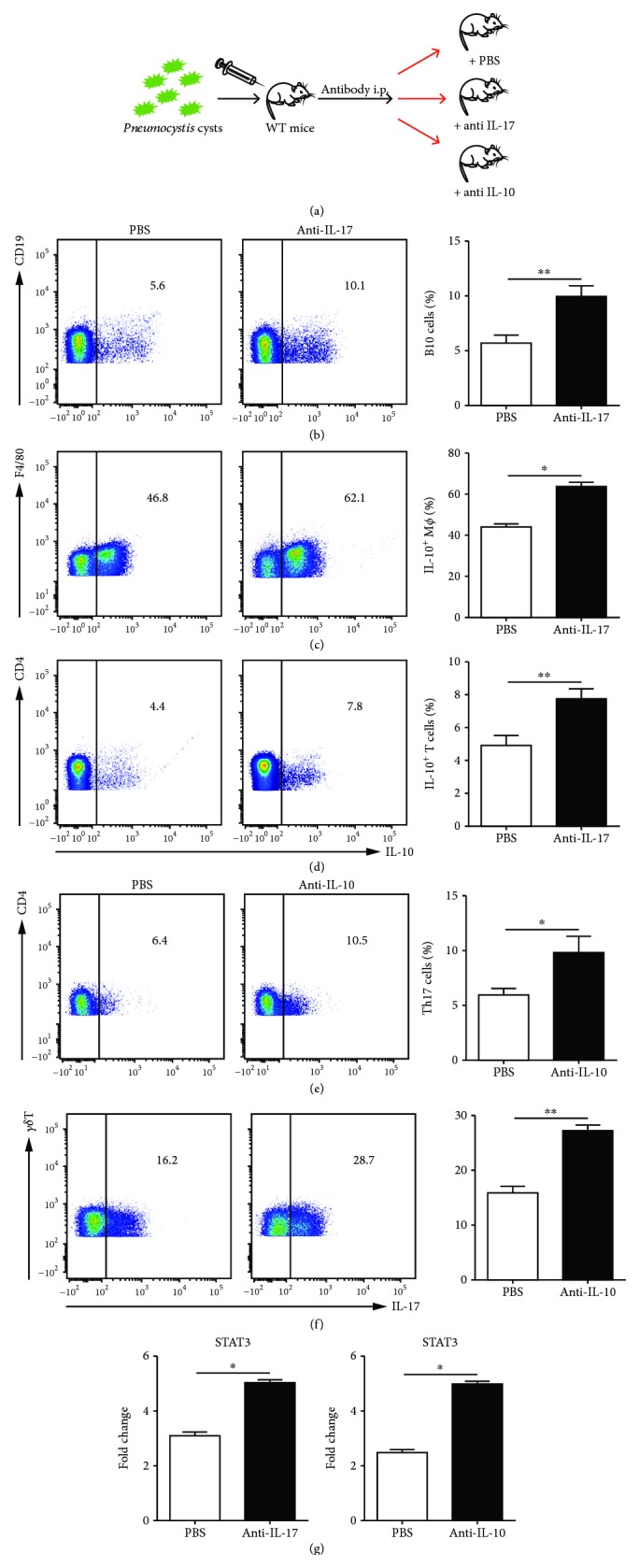
STAT3 was in association with the interactions of IL-17 and IL-10. The experimental design for the antibody neutralization is shown (a). Representative flow dot plots and comparisons of B10 cells (CD19^+^IL-10^+^) (b), IL-10^+^ macrophages (F4/80^+^IL-10^+^) (c), and IL-10^+^ T cells (CD4^+^IL-10^+^) (d) in the lung from WT PCP mice that received PBS or anti-IL-17 antibody. Representative flow dot plots and comparisons of Th17 cells (CD4^+^IL-17^+^) (e) and IL-17^+^*γδ*T cells (*γδ*T^+^IL-17^+^) (f) in the lung from WT PCP mice that received PBS or anti-IL-10 antibody. STAT3 gene expression in the lung from WT PCP mice the received anti-IL-17, anti-IL-10, or PBS was examined by RT-PCR (g). ^∗^*P* < 0.05, ^∗∗^*P* < 0.01, and ^∗∗∗^*P* < 0.001. Comparisons were evaluated by Student's *t*-test for two-group comparisons.

As STAT3 may play a role in the interplay of IL-17 and IL-10 in the PCP model, we performed IL-17 and IL-10 antibody neutralization experiments in *Pneumocystis*-infected mice (Figure 6(a)). We depleted IL-17 and IL-10 in WT PCP mice. The results showed that after injection of anti-IL-17 mAb, IL-10-expressing B cells (Figure 6(b)), macrophages (Figure 6(c)), and T cells (Figure 6(d)) were induced significantly in PCP mice. Also, after injection of anti-IL-10 mAb, the expression of STAT3 increased and Th17 cell (Figure 6(e)) and IL-17^+^*γδ*T cell (Figure 6(f)) percentages were higher in the lung from infected mice. Meanwhile, RT-PCR data demonstrated that depletion of IL-17 and IL-10 both promoted the expression of STAT3 (Figure 6(g)).

Thus, STAT3 may play an important role in the interactions of IL-17 and IL-10 in *Pneumocystis*-infected mice.

## 4. Discussion

Accumulating evidence indicates that PCP remains to be one of the most devastating diseases among non-HIV individuals receiving immunosuppressive therapy [1]. Multiple immune cells and cytokines have been studied in PCP hosts; however, it has been difficult to determine conclusively the cellular and molecular pathogenesis of PCP. Our present study focused on the immune regulatory roles of IL-17 and IL-10 in *Pneumocystis* pneumonia.

IL-17 is one of the founding members of the family of inflammatory cytokines, and IL-17 signaling is related to immunopathology and autoimmune diseases [17]. The proinflammatory role of IL-17 was demonstrated in host defense against pathogen in a number of chronic inflammatory diseases [18]. IL-17A and IL-17F act on various immune cells and increase the production of proinflammatory cytokines such as TNF-*α*, IL-1*β*, IL-6, and the granulocyte-macrophage colony-stimulating factor (GM-CSF) [29]. According to the related previous studies, IL-17 has two opposite contributions: its deficiency results in the loss of control of infections, while its overproduction could cause some chronic inflammatory diseases [18]. The study of Yen et al. demonstrated that IL-17^–/–^ mice are more susceptible to Staphylococcus aureus [30]. Awasthi and Kuchroo found that IL-17^–/–^ Candida albicans-infected mice show a higher fungal burden in skin lesion [31]. However, high levels of Th17 cells and CD8^+^ IL-17^+^ T cells were found in blood from patients with rheumatic diseases [32]. Meanwhile, the role of IL-17 in Crohn's disease remains unclear; IL-17 production leads to intestinal inflammation in several studies but could also be protective in others' researches [33, 34]. Thus, these data suggest that IL-17 could play a dual role in hosts defense against pathogens in chronic inflammatory diseases.

There are several studies focused on the immune function of IL-17 in the PCP model, but these results did not clarify the exact immune modulatory role of IL-17. Our present study showed that at 2 wk postinfection, IL-17 concentration was increased in serum and immune cells expressed more IL-17 in the lung from PCP mice. Our data is consistent with some studies from other investigators: Carmona et al. found that *β*-glucan surface components of *Pneumocystis* drive the activation of the IL-23/IL-17 axis, thus stimulating Th17 cell immunity in infected mice [13]; using a nude mouse model, Hu et al. verified that deficiency in IFN-*γ* promoted the differentiation of Th17 cells and IL-17 is essential for inflammatory responses in PCP [35]; Ripamonti et al. noted that IL-17^+^*γδ* T cells and CD4^+^ T cells in the lungs were increased during *Pneumocystis* infection in immunocompetent mice. However, the data of this study also demonstrated that IL-17A is not required for control of *Pneumocystis* infection [12], which is inconsistent with our present study. We found that the clearance of *Pneumocystis* was delayed in IL-17^–/–^ mice compared with WT mice. Likewise, depletion of IL-17 could not provide an experimental model for the formation of fungal-driven inducible bronchus-associated lymphoid tissue (iBALT), which is responsible for the *Pneumocystis* burden in the lung of infected mice [36]. The present study focused on the immune regulatory role of IL-17 in PCP hosts, and the results indicated that IL-17 levels elevated in infected individuals and it was essential in the clearance of *Pneumocystis*. Meanwhile, we found that depletion of IL-17 leads to the induction of IL-10 in the PCP model.

Interleukin-10 (IL-10) has long been recognized to be one of the vital anti-inflammatory cytokines, which has been unequivocally established in various models of infection, inflammation, and even cancer [20, 37]. IL-10^–/–^ mice could develop chronic inflammatory bowel disease [23]. In transgenic models, IL-10 reduced the ability of mice to mount significant T- or B-cell responses to ovalbumin, Listeria monocytogenes, and Leishmania [24]. Also, IL-10 expression constitutes a crucial element in the impairment of antiviral immunity [25]. According to these results, it is increasingly apparent that IL-10 might have a key role in inflammatory diseases. During *Pneumocystis* infection, IL-10 downregulates the immune response to pathogen in WT mice and plays an important role in controlling lung damage [38]. Furthermore, IL-10 was demonstrated to play a role in mediating B cell protection-demand hematopoiesis in PCP hosts [22]. In our previous study, we noted that B10 cells could play the immune regulatory role of Th1 and Th17 cell responses in infected mice [15]. In the current study, we further studied the immune modulatory role of IL-10. We noticed that IL-10 deficiency increased the proportion of the IL-17 level. These results suggested that during *Pneumocystis* infection, IL-17 inversely correlated with IL-10. In consistency with our data, some investigators also noted the interplay of IL-17 and IL-10 in inflammatory immunity. Mice lacking B10 cells were found to develop exacerbated disease and present with increased Th17 cell percentages [39]. Inhibiting IL-13 may inhibit Th17 production in an IL-10-dependent manner [40]. Mavropoulos et al. found that IL-10-producing B cells were impaired in psoriatic arthritis and inversely correlate with IL-17 and IFN-*γ* production [41]. Hansen et al. noted that IL-10 regulated an arthritic IL-17 response following infection with Borrelia burgdorferi [42]. These results indicated that IL-10 could play a significant role in the immune control and regulate the immune responses of the other cytokines during *Pneumocystis* infection.

Next, we detected the expression of IL-17-related genes in IL-17^–/–^ and IL-10^–/–^ PCP mice. Our data revealed the upregulation of STAT3 expression in IL-10^–/–^ PCP mice. Interestingly, IL-17-related genes were all downregulated in IL-17^–/–^ PCP mice except for the STAT3 gene. Thus, we suggested that the inverse correlation of IL-17 and IL-10 might be regulated via the STAT3 gene. STAT3 is one of the important transcription factors responsible for transmitting cytokine signals from the cellular membrane to the nucleus thus to alter gene expression, such as IL-6, type I and II interferon receptors, the IL-10 family receptors, and the IL-12 and IL-23 family receptors [43]. STAT3 activation is the downstream of a large number of cytokines via multiple receptor types [44, 45]. Recent evidences suggested a significant role of STAT3 in selectively maintaining a procarcinogenic inflammatory microenvironment [46]. In addition, STAT3 was reported to play a protective role in regulating virus-mediated proinflammation [47] and be associated with multiple immunodeficiency autoimmunity diseases [48]. Holland et al. found that mutations in the gene encoding STAT3 were identified in patients with autosomal dominant hyper-IgE syndrome (AD-HIES). Furthermore, the regulatory T cell and Th17 cell counts were reduced in these patients [49, 50]. Tangye et al. also suggested that STAT3 could play a critical part in the development of Th17 cells via affecting transcription of the genes encoding IL-17A, IL-17F, ROR*γ*T, and ROR*α* [51]. Meanwhile, activation of STAT3 is critical for IL-10 production [52]. These data and our results all indicated that STAT3 might play a key role in the inverse correlation of IL-17 and IL-10 in the PCP model.

In summary, all of the above demonstrated the pivotal roles of IL-17 and IL-10 in PCP hosts. IL-17 and IL-10 could both play protective roles in *Pneumocystis* infection via attenuating lung damage and assisting the clearance of pathogen. In addition, IL-17 and IL-10 inversely correlated with each other in the PCP model. We also noted that STAT3 might play a central role in the interplay of IL-17 and IL-10 during infection and it may be a new target for the therapy of PCP in the future.

## Data Availability

The data used to support the findings of this study are available from the corresponding author upon request.

## Supplementary Materials

Supplementary Materials 1. Supplementary Fig. 1: comparisons of periodic acid silver methenamine-stained histological features of the lungs of WT mice (A) and WT-PCP mice (B) at 2 wk postinfection. 2. Supplementary Fig. 2: IL-17 and IL-10 levels in serum from Pneumocystis-infected mice. Representative flow cytometric dot plots and comparisons of Th17 (CD4^+^IL-17^+^) cells (A) and IL-17^+^*γδ*T (*γδ*T^+^IL-17^+^) cells (B) in the blood from PCP mice and WT mice. Representative flow cytometric dot plots and comparisons of B10 cells (CD19^+^IL-10^+^) (C) and IL-17^+^CD4^+^ T cells (CD4^+^IL-10^+^) (D) in blood from PCP mice and WT mice. Comparisons were evaluated by Student's *t*-tests for two-group comparisons. ^∗^*P* < 0.05. Con: control; PCP: Pneumocystis pneumonia. 3. Supplementary Fig. 3: IL-17 deficiency did not influence the percentages of CD4^+^ T cells, CD8^+^ T cells, B cells, and macrophages in the lung from PCP mice. Representative flow dot plots and comparisons of CD4^+^ T cells (CD3^+^CD8^−^), CD8^+^ T cells (CD3^+^CD8^+^) (A), B cells (CD3^−^CD19^+^) (B), and macrophages (CD45^+^F4/80^+^) (C) in the lungs from WT PCP mice and IL-17^–/–^ PCP mice. Comparisons were evaluated by Student's *t*-test for two-group comparisons. 4. Supplementary Fig. 4: IL-10 deficiency influenced the percentages of B cells and *γδ*T cells in the lung from PCP mice. Representative flow dot plots and comparisons of CD4^+^ T cells (CD3^+^CD8^−^), CD8^+^ T cells (CD3^+^CD8^+^) (A), B cells (CD3^−^CD19^+^) (B), and *γδ*T cells (CD3^+^*γδ*T^+^) (C) in the lungs from WT-PCP mice and IL-10^–/–^ PCP mice. Comparisons were evaluated by Student's *t*-test for two-group comparisons.
