# Supplementary material for: IL-17 Inversely Correlated with IL-10 via the STAT3 Gene in Pneumocystis-Infected Mice
Source: Mediators Inflamm. 2019 Sep 10;2019:6750861. doi: 10.1155/2019/6750861 (PMC6754930; doi:10.1155/2019/6750861)

Supplement for full details of methods and additional figure

Methods

PCP models and sample processing

Primers and probes for the *P.*murina RNA were as follows: 5’-AGG TGA AAA GTC GAA AGG GAA AC-3’ and 5’-AAA ACC TCT TTT CTT TCA CTC AGT AAC A-3’; 5’-FAM-CCC AGA ATA ATG AAT AAA G-MGBNFQ-3’.

Flow cytometry

The antibodies included Anti-human CD3, CD19, IL-17, IL-10 and Anti-mouse CD3, CD8, CD4, CD19, B220, γδT, IFN-γ, IL-4, IL-17, IL-10 which were purchased from BD Pharmingen (San Diego, CA, USA) and eBioscience (San Diego, CA, USA).

Flow cytometry for surface markers and for intracellular detection of cytokines was performed as described previously. The flow cytometry data were analyzed using BD FCS Diva Software and FCS Express 5 software (De Novo Software, Los Angeles, CA).

Real-Time PCR

Primers and probes for validation of IL-17 related genes were as follows: STAT3 5’-CAC CTT GGA TTG AGA GTC AAG AC-3’ and 5’-AGG AAT CGG CTA TAT TGC TGG T-3’, STAT5 5’-CGA TGC CCT TCA CCA GAT G-3’ and 5’-AGC TGG GTG GCC TTA ATG TTC-3’, RORγT 5’-GAC CCA CAC CTC ACA AAT TGA-3’ and 5’-AGT AGG CCA CAT TAC ACT GCT-3’, IFN-γ 5’-ATG AAC GCT ACA CAC TGC ATC-3’ and 5’-CCA TCC TTT TGC CAG TTC CTC-3’, STAT1 5’-TCA CAG TGG TTC GAG CTT CAG-3’ and 5’-GCA AAC GAG ACA TCA TAG GCA-3’， GATA3 5’-CTC GGC CAT TCG TAC ATG GAA-3’ and 5’-GGA TAC CTC TGC ACC GTA GC-3’, Irf4 5’-TCC GAC AGT GGT TGA TCG AC-3’ and 5’-CCT CAC GAT TGT AGT CCT GCT T-3’.

Supplementary Fig.1 Comparisons of periodic acid-silver methenamine stained histological features of the lung of WT mice(A) and WT-PCP mice(B) at 2 wk post-infection.

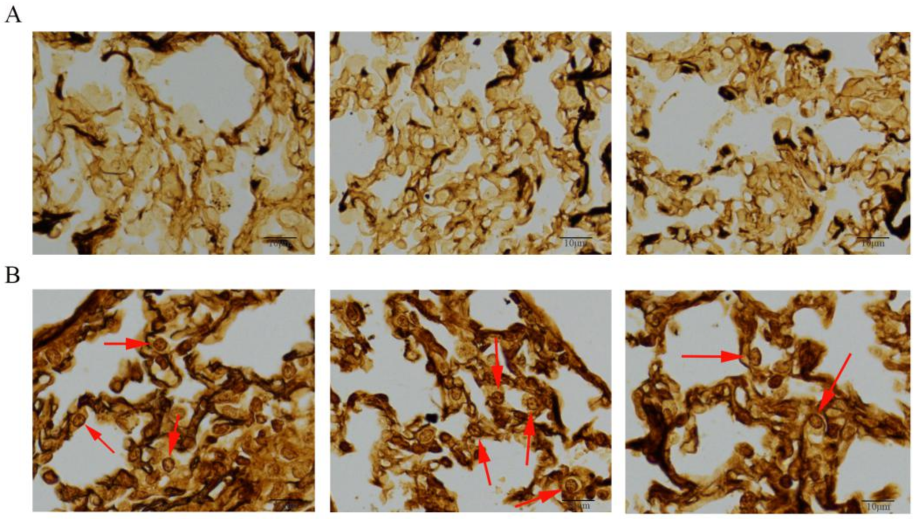

Supplementary Fig.2 IL-17 and IL-10 levels in serum from *Pneumocystis*-infected mice. Representative flow cytometric dot plots and comparisons of Th17($CD4^+IL\text{-}17^+$) cells (A) and IL-17^+ γδT (γδT^+IL-17^+) cells (B) in the blood from PCP mice and WT mice.Representative flow cytometric dot plots and comparisons of B10 cells ($CD19^+IL\text{-}10^+$, C) and IL-17^+CD4^+ T cells ($CD4^+IL\text{-}10^+$, D) in blood from PCP mice and WT mice. Comparisons were evaluated by Student's t-tests for two-group comparisons. $*P < 0.05$. Con, control; PCP, *Pneumocystis* pneumonia.

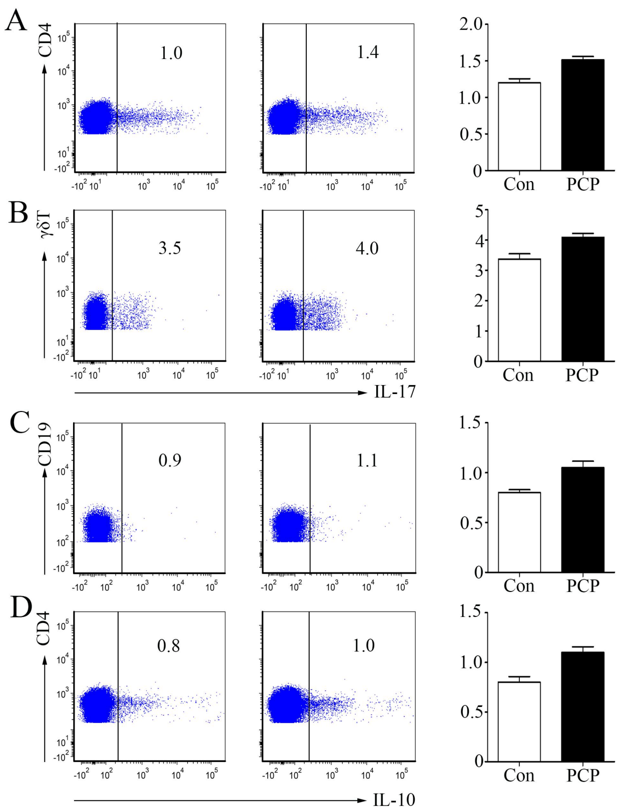

Supplementary Fig.3 IL-17 deficiency did not influence the percentages of $CD4^+$ T cells, $CD8^+$ T cells, B cells and macrophages in lung from PCP mice. Representative flow dot plots and comparisons of $CD4^+$ T cells ($CD3^+CD8^-$), $CD8^+$ T cells($CD3^+CD8^+$)(A), B cells ($CD3^-CD19^+$, B) and Macrophages ($CD45^+F4/80^+$,C) in the lung from WT PCP mice and IL-$17^{-/-}$ PCP mice. Comparisons were evaluated by Student's t test for two-group comparisons.

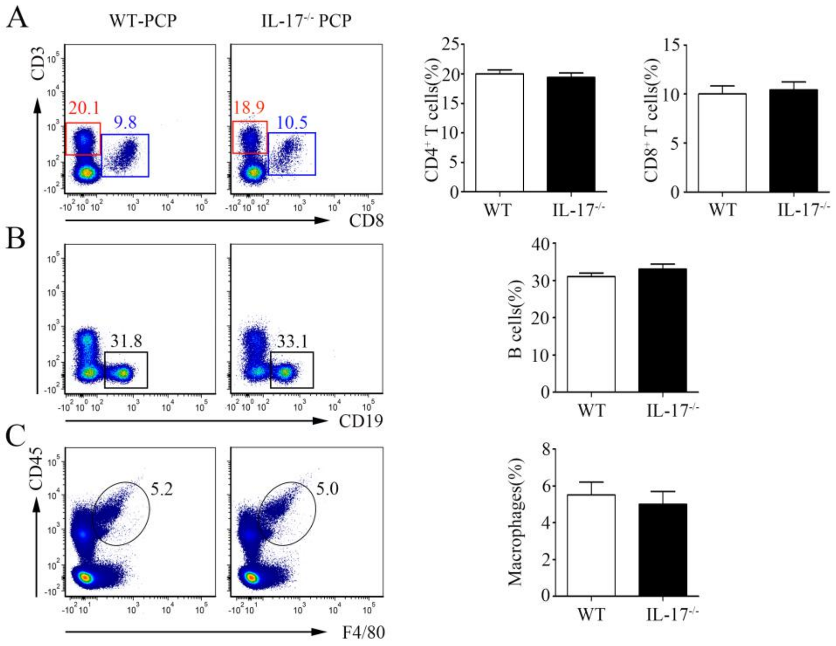

Supplementary Fig.4 IL-10 deficiency influenced the percentages of B cells and γδT cells in lung from PCP mice. Representative flow dot plots and comparisons of $CD4^+$ T cells ($CD3^+CD8^-$), $CD8^+$ T cells ($CD3^+CD8^+$) (A), B cells ($CD3^-CD19^+$, B) and γδT cells ($CD3^+γδT^+$, C) in lung from WT-PCP mice and $IL\text{-}10^{-/-}$ PCP mice. Comparisons were evaluated by Student's t test for two-group comparisons.

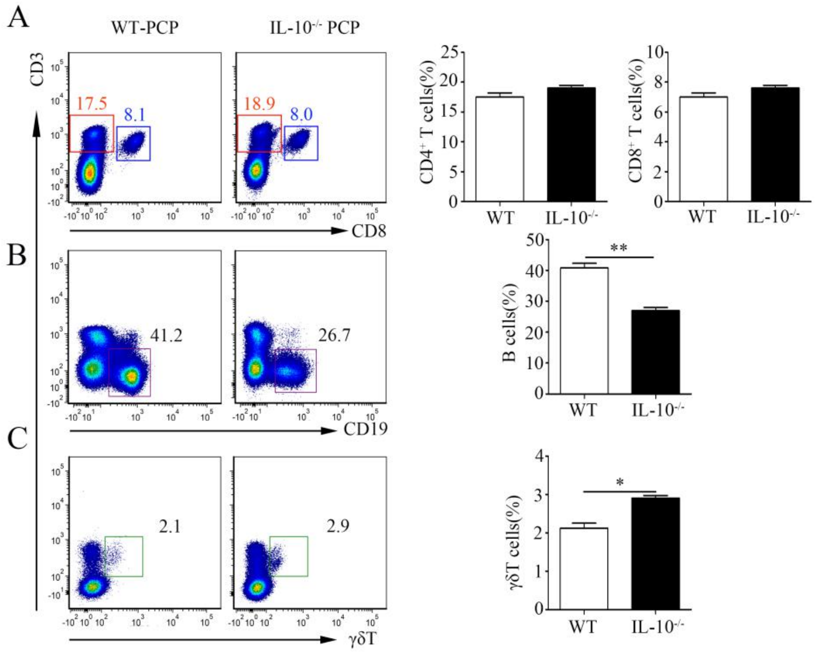

Supplement: Supplementary Materials — 1. Supplementary Fig. 1: comparisons of periodic acid silver methenamine-stained histological features of the lungs of WT mice (A) and WT-PCP mice (B) at 2 wk postinfection. 2. Supplementary Fig. 2: IL-17 and IL-10 levels in serum from Pneumocystis-infected mice. Representative flow cytometric dot plots and comparisons of Th17 (CD4+IL-17+) cells (A) and IL-17+γδT (γδT+IL-17+) cells (B) in the blood from PCP mice and WT mice. Representative flow cytometric dot plots and comparisons of B10 cells (CD19+IL-10+) (C) and IL-17+CD4+ T cells (CD4+IL-10+) (D) in blood from PCP mice and WT mice. Comparisons were evaluated by Student's t-tests for two-group comparisons. ∗P < 0.05. Con: control; PCP: Pneumocystis pneumonia. 3. Supplementary Fig. 3: IL-17 deficiency did not influence the percentages of CD4+ T cells, CD8+ T cells, B cells, and macrophages in the lung from PCP mice. Representative flow dot plots and comparisons of CD4+ T cells (CD3+CD8−), CD8+ T cells (CD3+CD8+) (A), B cells (CD3−CD19+) (B), and macrophages (CD45+F4/80+) (C) in the lungs from WT PCP mice and IL-17–/– PCP mice. Comparisons were evaluated by Student's t-test for two-group comparisons. 4. Supplementary Fig. 4: IL-10 deficiency influenced the percentages of B cells and γδT cells in the lung from PCP mice. Representative flow dot plots and comparisons of CD4+ T cells (CD3+CD8−), CD8+ T cells (CD3+CD8+) (A), B cells (CD3−CD19+) (B), and γδT cells (CD3+γδT+) (C) in the lungs from WT-PCP mice and IL-10–/– PCP mice. Comparisons were evaluated by Student's t-test for two-group comparisons. [file 6750861.f1.pdf]
